# Content of Toxic Elements in 12 Groups of Rice Products Available on Polish Market: Human Health Risk Assessment

**DOI:** 10.3390/foods9121906

**Published:** 2020-12-20

**Authors:** Joanna Bielecka, Renata Markiewicz-Żukowska, Patryk Nowakowski, Monika Grabia, Anna Puścion-Jakubik, Konrad Mielcarek, Krystyna Joanna Gromkowska-Kępka, Jolanta Soroczyńska, Katarzyna Socha

**Affiliations:** Department of Bromatology, Medical University of Białystok, Mickiewicza 2D Street, 15-222 Białystok, Poland; renmar@poczta.onet.pl (R.M.-Ż.); patryk.nowakowski@umb.edu.pl (P.N.); monika.grabia@umb.edu.pl (M.G.); anna.puscion-jakubik@umb.edu.pl (A.P.-J.); konrad.mielcarek@umb.edu.pl (K.M.); krystyna.gromkowska.kepka@gmail.com (K.J.G.-K.); jolanta.soroczynska@umb.edu.pl (J.S.); katarzyna.socha@umb.edu.pl (K.S.)

**Keywords:** rice, rice products, pigmented rice, heavy metals, quality evaluation, health risk assessment

## Abstract

Background: Rice is one of the most commonly consumed grains. It could be a good source of nutrients in a diet, but its consumption could also contribute to exposure to toxic elements. All rice products available on the Polish market are imported, which may pose a particular concern as to the safety of their consumption. The aim of our study was to estimate the content of As, Cd, Pb, and Hg in rice products and to assess the health risk indicators related to exposure to toxic elements consumed with rice products among the adult population in Poland. Methods: A total of 99 samples from 12 groups of rice products (basmati, black, brown, parboiled, red, wild, white rice and expanded rice, rice flakes, flour, pasta, and waffles) available in the Polish market were obtained. The content of Hg was determined using the atomic absorption spectrometry method (AAS). To measure As, Cd, and Pb, inductively coupled plasma-mass spectrometry (ICP-MS) was used. The health risk was assessed by calculating several indicators. Results: The average As, Cd, Pb, and Hg contents in all studied products were 123.5 ± 77.1 μg/kg, 25.7 ± 26.5 μg/kg, 37.5 ± 29.3 μg/kg, and 2.8 ± 2.6 μg/kg, respectively. Exceedance of the limit established by the Polish National Food Safety Standard was observed in one sample as regards the As content and exceedance of the European Commission standard in two samples for Hg. The samples of foods imported from European markets (*n* = 27) had statistically higher As content (*p* < 0.05) than those imported from Asian countries (*n* = 53). The values of health risk indicators did not show an increased risk for the Polish adult population. However, the daily intake of 55 g of rice corresponds to the benchmark dose lower confidence limit (BMDL) for Pb. Conclusion: The studied rice products could be regarded as safe for consumption by the Polish population as far as the content of As, Cd, Pb, and Hg is concerned.

## 1. Introduction

Besides wheat and maize, rice is one of the most commonly grown and consumed grains. This cereal is a staple food in many countries around the world, especially in Asia, but its consumption outside Asia has increased in recent years. According to the Food and Agriculture Organization data, in the years 2015–2017 world rice production reached 498.3 million (mln) metric tons, of which 403.4 mln metric tons was used for food. It is assumed that over the next years, world rice production will continuously increase to 562.3 mln metric tons (in 2027) and that rice consumption will be higher as well — 459.5 mln metric tons. More than 90% of the rice available on the world market is cultivated in Asian countries, whereas European rice crops constitute only 0.6%. Among the countries with the greatest rice production, China, India, and Indonesia are listed. It is predicted that in 2027, in Europe there will be an increase in rice consumption by 0.66% in comparison with data from 2008 to 2017 [1]. Taking into account the available global statistics, the average rice consumption between 2017 and 2018 was estimated at 53.8 kg per capita per year (4.86 kg per month) [2]. Meanwhile, in Poland average monthly rice consumption per capita in 2018 was estimated at 0.15 kg [3].

The most commonly cultivated rice species is *Oryza sativa* L. (Asian rice), followed by *Oryza glaberrima* S. (African rice). Only slight morphological differences separate these two varieties. Rice is cultivated in many different regions all around the world. The content of nutrients in rice is dependent on the type of soil and the environmental conditions in which it grows [4]. This grain has a high ability to accumulate As and Cd from the soil, and their content in rice depends on growth conditions. Rice is generally grown as a lowland crop in flooded soil under reducing conditions that greatly contribute to excessive As bioaccumulation. On the other hand, aerobic treatment decreases As but increases Cd accumulation in rice grains [5]. Some other metals (e.g., Pb and Hg) have very low solubility in soil and therefore plants absorb very small amounts of them [6]. The steps of processing the grain are dependent on the end product and include drying of the grain, removal of the husk, and milling to remove the bran layer, which is used for white rice production [4]. In addition, in parboiled rice the grain is first soaked in hot water, then steamed before drying. Unpolished grains (whole grains with the germ, bran, and endosperm) are rich in fiber, vitamins— especially B vitamins (thiamine, pantothenic acid, folate) and vitamin E—and minerals such as iron, calcium, and zinc. The polishing process removes the bran and the germ, consequently decreasing the mineral content and hence the nutritional value of rice [7].

The health-promoting and antioxidant properties of selected rice species vary across the varieties. The color of the red and the black (purple) grain species is due to the presence of anthocyanins and proanthocyanidins in the pericarp and aleurone layers. Naturally pigmented species have a greater content of bioactive compounds, such as anthocyanins, tocopherols, phenolic compounds, γ-oryzanols, tocotrienols, phytosterols, and phytic acid compared to non-pigmented ones. Therefore, these grains have a higher nutritional value than white varieties. It was demonstrated that extracts of pigmented rice neutralized reactive oxygen species due to the content of phenolic compounds and anthocyanins [8]. It is important that the health-promoting properties of individual varieties of rice should not be negatively affected by toxic elements. Therefore, it seems crucial to control the content of toxic elements due to their possible negative impact on the bioavailability of nutrients and the risk for human health.

Toxic elements, which are also defined as heavy metals, include metals and metalloids with an atomic density higher than 5 g/cm^3^ and could have a negative influence on living organisms and the environment [9]. Exceedance of certain threshold concentrations could cause many adverse health effects, considering their tendency for accumulation over time, poor biodegradability, and long biological half-lives. This group comprises, among others, arsenic (As), cadmium (Cd), lead (Pb), and mercury (Hg). As, Cd, Pb, and Hg are classified in the top 10 of the list created by the Agency for Toxic Substances and Disease Registry (ATSDR), based on the combination of frequency, toxicity, and potential health effects of exposure for humans [10]. Heavy metals are present in the environment due to their natural occurrence (e.g., soil erosion) as well as due to anthropometric emissions. Their toxic outcomes on health depend on the dose and duration of exposure and primarily affect the functioning of the brain, liver, kidneys, and lungs. Numerous long-term impacts of heavy metals on the occurrence of diseases such as Alzheimer’s, Parkinson’s, and multiple sclerosis are described [9].

As commonly occurs in both organic (As) and inorganic forms (iAs)—as arsenate As(V)/iAs(V) or arsenite As(III)/iAs(III). These forms are also the most prevalent in land-grown foods. This metalloid can be transferred to food mainly through contaminated water and soil. Among the principal routes of As exposure by humans, food and drinking water are mentioned [11]. The iAs form has significantly higher toxicity and is retained in the body longer compared to organic As. In addition, As and iAs compounds have been categorized by the International Agency for Research on Cancer (IARC) as group 1 carcinogens with sufficient evidence in humans [12]. Exposure to iAs may lead to the development of several cancers, such as skin, kidney, lung, liver, or bladder cancer [12].

Cd and its compounds are also categorized by IARC as group 1 carcinogens [12]. Exposure to this toxic element is associated with increased risk of numerous cancers, such as breast, endometrium, lung, and bladder cancer [13]. Among the main routes of Cd exposure for the smoking population, cigarettes are mentioned, while for non-smokers, dietary intake of Cd is responsible for 90% of exposure. Only a relatively small percentage of ingested Cd is absorbed from the digestive tract, but the very long biological half-life of this heavy metal (ranging from 10 to 30 years) is cause for considerable concern. The kidneys, as well as the bones, are particularly vulnerable to Cd toxicity [13].

The risk of negative health effects has also been demonstrated for Pb. The IARC regards Pb as a class 2A carcinogen, which means that it can probably contribute to the development of cancer in humans [12]. The main routes of exposure to this toxic element include food, water, air, and soil. Chronic exposure to low doses of Pb is concerning, because lead has the ability to accumulate in the body, in particular in the skeletal system. The central nervous system is the most critical target of Pb toxicity, especially during brain development. In addition, this heavy metal could impair the functioning of several other systems in the body, such as the immune, reproductive, endocrine, gastrointestinal, cardiovascular, and renal systems [14].

Three chemical forms of Hg are distinguished: metallic (Hg^0^), inorganic (Hg_2_^2+^, Hg_2_), and organic Hg (methylmercury (MeHg), dimethylmercury, ethylmercury, phenylmercury). Each of these forms has different bioavailability and exhibits various toxic effects. MeHg has greater toxicity for humans as well as higher absorption from food (95%) compared to iHg (nearly 8%). MeHg is considered to be the most toxic form of organic Hg. The target organ of the toxic influence of Hg is the brain; however, it can also impair the functions of the nervous, renal, and muscular systems [15]. Nevertheless, reports from recent years have demonstrated that rice grains could also contribute to MeHg exposure from the diet [16]. Plant-based foods are regarded as the main sources of toxic elements in the human diet. Various indicators have been designed that can be used to estimate exposure to toxic elements from the diet. To estimate short-term exposure, the following can be used: estimated daily intake (EDI), estimated weekly intake (EWI), provisional tolerable weekly intake (PTWI), provisional tolerable monthly intake (PTMI). Indicators used to assess the risk of long-term negative health effects include the benchmark dose lower confidence limit (BMDL), target hazard quotient (THQ), hazard index (HI), and cancer risk (CR). The selection of an appropriate indicator for a specific element should be based on available literature data regarding its impact on the body. The majority of the available research into human health outcomes of exposure to toxic elements as a result of rice consumption was conducted in Asian countries. Rice species are not cultivated in Poland due to unfavorable climate conditions. All of the rice consumed by Poles is imported, mainly from Asia, South America, Africa, and Southern Europe. Moreover, there is a lack of a comprehensive assessment of exposure to toxic elements with diet in the Polish population. Therefore, there is a need to investigate the content of toxic elements in rice and to assess the safety of consumption of rice products available on the Polish market, which has not been previously addressed. The novel approach of our investigation also involves the measurement of toxic element content in a broad range of rice species, with particular attention to rice products such as pasta, flakes, flour, waffles, and expanded rice, which have not yet been studied in this aspect.

The aim of this study was to estimate the contents of toxic elements (As, Cd, Pb, Hg) in rice samples as well as rice products available on the Polish market and to compare them across different subgroups of products, considering the country of origin. Furthermore, the health risk among the adult Polish population, as well as the risk of selected populations (where rice is produced) resulting from the intake of toxic elements from rice products, was assessed.

## 2. Materials and Methods

### 2.1. Sample Collection

The samples (*n* = 99) were purchased in standard packaging available locally in northeastern Poland between March and May 2020. Our sampling strategy aimed to collect as many different subgroups with representative samples as possible. We obtained the following research material: rice flakes (12), white rice (11), basmati rice (10), parboiled rice (10), brown rice (10), rice waffles (9), expanded rice (8), rice pasta (7), rice flour (6), black rice (6), red rice (5), and wild rice (5). Wild rice (*Zizania* sp.), which is not classified in the rice family, was included in the research due to its similarity to *Oryza* spp. Among the subgroups above we chose the products to be studied (a minimum of five samples), each sample in the subgroup being from a different producer, without replication. The samples were representative of the whole Polish market. The largest number of samples was imported from Thailand—18, followed by 16 from Italy, 12 from Pakistan, 9 from Burma, 6 from India, 5 from Cambodia, 3 from Vietnam, 2 from Belgium, and one each from the following countries: Brazil, United States of America, Canada, Bulgaria, Spain, France, and Holland. Out of all the samples collected, 19 producers did not reveal the country of origin of the products.

### 2.2. Sample Digestion

Whole grains were not rinsed or cooked prior to being prepared for analysis. All samples were homogenized in a stainless-steel mill before digestion, weighed (0.2–0.3 g), and placed in mineralization polytetrafluoroethylene vessels. Then 4 mL of spectrally pure concentrated (69%) HNO_3_ was added (Tracepur, Merck, Darmstadt, Germany). Microwave digestion was performed in a closed-loop system (Berghof, Speedwave, Eningen, Germany). The overall process consisted of four steps, presented in Table 1. After mineralization, the samples were quantitatively transferred to polypropylene vessels and then diluted 10 times. The content of As, Cd, and Pb was recalculated and shown as µg/kg of product.

### 2.3. Toxic Elements Analysis

#### 2.3.1. Arsenic, Cadmium, and Lead

Inductively coupled plasma-mass spectrometry (ICP-MS, NexION 300D, PerkinElmer, USA) with a kinetic energy discrimination (KED) chamber was used in the case of As, and in the standard mode in the case of Cd and Pb. Kinetic energy discriminations and collisions were used for correcting polyatomic interferences in this configuration. The results were obtained in counts per second (cps) and based on calibration curves, were converted into concentrations. To determine the limit of detection (LOD), 10 independent blank determinations were made. A three-fold standard deviation (SD) from the mean value determined in concentration units was taken as the LOD. The LOD values were 0.019 μg/kg for As, 0.017 μg/kg for Cd, and 0.16 μg/kg for Pb. A detailed description of the parameters on the basis of which the determinations were carried out is presented in Table 2.

#### 2.3.2. Mercury

The determination of Hg content did not require a mineralization process. Hg content was determined by the atomic absorption spectrometry method (AAS) using the amalgamation technique (AMA-254, Leco Corp., Altec Ltd., Prague, Czech Republic). The samples, weighed (0.12–0.15 g) with an accuracy of 1 mg, were placed in a cuvette and analyzed. The first phase lasted 60 s and the samples were dried and burned in oxygen at 600 °C. Then the vapors of mercury passed the catalytic column and were collected by the amalgamator; the process took 150 s. The last phase lasted 45 s: Hg was released from the amalgamator and was measured by atomic absorption spectrometry at a wavelength of 245 nm. The limit of detection was 0.003 ng per sample.

#### 2.3.3. Quality Control

Quality control was performed by analyzing certified reference material (corn flour INCT-CF-3, Institute of Nuclear Chemistry and Technology, Warsaw, Poland) prior to the start of the analysis and every 10 samples. The results of the quality control are summarized in Table 3.

### 2.4. Health Risk Assessment

The risk of adverse health effects resulting from the intake of the studied chemicals from rice consumption was assessed by calculating for each element selected indicators such as the estimated daily intake (EDI), the estimated weekly intake (EWI), the target hazard quotient (THQ), and the hazard index (HI). The THQ is described as the ratio of exposure to a toxic element and the reference dose, which is the highest level at which no negative health effects are expected. Reference doses were established at specific levels for individual trace elements. The HI shows cumulative exposure to several potentially toxic elements, which may be important when separate THQs calculated for individual elements show no potential risk of adverse health effects. THQ and HI were used to estimate non-cancer hazard risk, whereas cancer risk (CR) was calculated in a separate equation. As recommended by the Joint FAO/WHO Expert Committee on Food Additives (JECFA), different indicators were used for the studied elements. For Hg and Cd, the provisional tolerable weekly intake (PTWI) and the provisional tolerable monthly intake (PTMI) were estimated, respectively. For As and Pb, the benchmark dose lower confidence limits (BMDL) were identified. We determined these indicators in order to evaluate the possible risk of short- and long-term adverse health effects. All the indicators were calculated based on the following equations:

EDI = (C × Cons)

where C is the concentration of a given heavy metal in analyzed rice and Cons is the average daily consumption of rice in Poland and in selected countries.

EWI was calculated by multiplying the EDI by 7, corresponding to 7 days.

PTWI = EDI × 7/BW

where BW is the average body weight (kg). The PTWI value for Hg was assumed, in line with JECFA guidelines, as 4 µg/kg BW/week [17].

PTMI = EDI × 30/BW

where the JECFA reference for Cd was 25 µg/kg BW/month [17].

BMDL = EDI/BW

According to the JECFA, the reference BMDL_0.5_ for As is 3 µg/kg BW/day (the benchmark dose lower confidence limit for a 0.5% increased incidence of lung cancer) and for Pb is 0.02–3 µg/kg BW/day [17]. BMDL values determine the lowest doses associated with the development of a specific effect on the human body.

THQ = (Fr × D × Cons × C)/(RfD × BW × T) × 10^−3^

where Fr is the frequency of exposure (365 days/year), D is the duration of exposure (the average lifetime of 70 years), Cons is the average rice consumption per day (g/day), C is the concentration of the studied elements in the samples (mg/kg), and RfD is the oral reference dose, determined by the United States Environmental Protection Agency (US EPA) for As as 0.3 µg/kg BW/day, for Cd and Pb as 1 µg/kg BW/day, and for Hg as 0.3 µg/kg BW/day [18]. T is the overall time of exposure for non-carcinogens (365 days/year × 70 years).

If the value of the THQ is >1, it may suggest a potential risk due to the intake of a given heavy metal from rice consumption. When the value is <1, it could be assumed that there is a low risk of a non-cancerogenic effect.

HI was calculated as a sum of the individual THQ calculated to assess the additive effect of all the pollutants. The reference point in our study was <4. A higher value would mean a risk of negative health effects.

CR = (Fr × D × EDI × Sf)/T x 10^−3^

where Sf is the slope factor for chemicals known as carcinogens and estimates the probability that an individual will develop cancer if exposed for a lifetime of 70 years. The other individual components of this equation are described above. According to the US EPA, the values of Sf were adopted as follows: for As—1.5 mg/kg/day, for Cd—6.3 mg/kg/day, and for Pb—0.0085 mg/kg/day [18]. CR indicates the probability of developing cancer. A value of CR higher than 10^−4^ means an increased risk of a carcinogenic effect. CR was not evaluated for Hg because, according to the IARC, Hg is not considered to be a potentially carcinogenic element.

The average daily rice consumption in Poland, based on the data above [3], is estimated at 5 g/day for adults. We assumed 70 kg as the average body weight. However, in the health risk assessment for adults, we considered an alternative to the highest permissible per capita consumption of rice in the Polish population. Based on our results and with regard to the established limits, we calculated the level of consumption that may be related to negative health effects. Due to the fact that the largest number of samples (18) had been imported from Thailand, we also assessed the health risk for this population. For Thai adults, unlike for the Polish population, rice is a staple food and its consumption ranges from 80 to 155 kg per capita per year [19]. To calculate the health risk indicators, the average daily consumption was assumed to be 322 g and 60 kg was taken as the average body mass of a Thai person [20]. This assessment was conducted to learn whether the imported products that we regarded as representative samples from Thailand available on the Polish market could pose a health hazard to Thai residents in terms of the measured content of toxic elements.

### 2.5. Data Analyses

The data were analyzed using the Statistica software (TIBCO Software Inc., Palo Alto, CA, USA). The Shapiro–Wilk test was used to assess the conformity of data distribution with normal distribution. Due to the lack of normality in the distribution of data, to compare the content of heavy metals considering the type of product as well as the country of origin, the non-parametric Mann–Whitney U-test and the Kruskall–Wallis Analysis of Variance (ANOVA) were performed. To describe the content of the studied elements in rice and rice products, the median (Me) and quartiles (Q) were used. Additionally, to make it easier to compare our results with those obtained by other researchers, the mean (X), standard deviation (SD), minimum (Min), and maximum (Max) were added in the tables. The correlation of Spearman with the Bonferroni correction was used to check the relationship between the content of the elements tested in all product subgroups. Significant difference values were assumed at *p* < 0.05, *p* < 0.01, and *p* < 0.001. Considering the country of origin, we conducted the analysis taking into account the division into rice imported from Asian (*n* = 53) and European (*n* = 27) markets.

## 3. Results

### 3.1. Content of As, Cd, Pb, and Hg

Table 4 contains the levels of heavy metals in the studied rice samples and rice products. In our investigation, the mean As content in all the analyzed products was found to be 123.5 ± 77.1 µg/kg (*n* = 99), with the lowest content (54.6 ± 6.3 µg/kg) determined in pasta (*n* = 7) and the highest (252.2 ± 173.6 µg/kg) in red rice samples (*n* = 5). In contrast, in individual products, the lowest As level was detected in the sample of black rice produced in Thailand (4.2 µg/kg) and the highest in black rice imported from Europe (562.2 µg/kg). The regulations of the European Commission (EC) determining the standards of maximum levels of certain contaminants in foodstuffs provide the guidelines for iAs content [21]. The speciation of iAs was not performed in our research, therefore the Polish National Food Safety Standard (PNFSS) for total As content in foodstuffs was used [22], which set the maximum level of total As at 500 µg/kg. In our research, one sample exceeded this limit.

The average level of Cd in all the examined products was measured at 25.7 ± 26.5 µg/kg. Taking into account the individual subgroups of products, the greatest Cd content was detected in flour (*n* = 6, 50.2 ± 21.8 µg/kg), whereas basmati rice samples had the lowest Cd level (*n* = 10, 14.2 ± 15.2 µg/kg). However, among the individual products, the highest Cd was found in parboiled rice imported from Thailand (171.6 µg/kg), whereas the lowest was in flakes produced in Europe (0.3 µg/kg). The EC standards of Cd levels in rice was established at 200 µg/kg [23]. No exceedance of the limits was revealed.

Next, the mean Pb content in all samples (*n* = 99) was assessed and was found to be 37.5 ± 29.3 µg/kg. The highest Cd levels were observed in expanded rice products (*n* = 8, 79.2 ± 36.8 µg/kg), whereas the lowest was in flakes (*n* = 12, 18.4 ± 13.3 µg/kg). As in the case of Cd, the lowest content of Pb was identified in one sample of flakes from the European market (0.4 µg/kg), and the highest in expanded rice (135.6 µg/kg). According to the EC regulations, the maximum permissible Pb content in grains is 200 µg/kg. No exceedance of the limit was noted in any product [23].

The last toxic element we studied was Hg. Its average content in all the products was 2.8 ± 2.6 µg/kg. The highest mean Hg level was detected in parboiled rice (*n* = 10, 4.0 ± 5.0 µg/kg), whereas the lowest was in flour (1.8 ± 0.8 µg/kg). However, two individual parboiled rice products proved to have the lowest (0.1 µg/kg, produced in Cambodia) as well as the highest (15.8 µg/kg, imported from India) Hg content observed. The EC regulation prescribes that the maximum level of total Hg in rice is 10 µg/kg [24]. Two samples were recorded to exceed the acceptable standard.

Table 5 presents significant differences in the content of the studied elements across the particular subgroups, detected in statistical analyses. The highest number of statistically significant differences regarded the content of arsenic. The measured content of As in pasta samples differed from the content of As in brown and red rice (*p* < 0.05). Additionally, the content of As in basmati rice was significantly different from the content of As in red (*p* < 0.05) and brown rice (*p* < 0.001). Significant differences between the content of As in waffles and pasta were also discovered (*p* < 0.001). As far as Cd content is concerned, the conducted statistical analyses showed that it differed between the flour and basmati rice subgroups (*p* < 0.05). With regard to Pb levels in the measured samples, a significant difference was found between white and expanded as well as red rice (*p* < 0.05) and between flakes and expanded rice products (*p* < 0.001). On the other hand, considering Hg concentration, no significant differences were found among the analyzed subgroups.

In the subgroup analysis, in white rice and in basmati rice siginificant correlations between Cd and Pb (r = 0.78, *p* < 0.05) were observed. In expanded rice as well as in black rice, significant correlations (r = −0.74, *p* < 0.05) for Cd and As were detected. Additionally, a significant correlation was found between Pb and Hg (r= −0.66, *p* < 0.05) in the parboiled rice samples.

**Figure 1:**
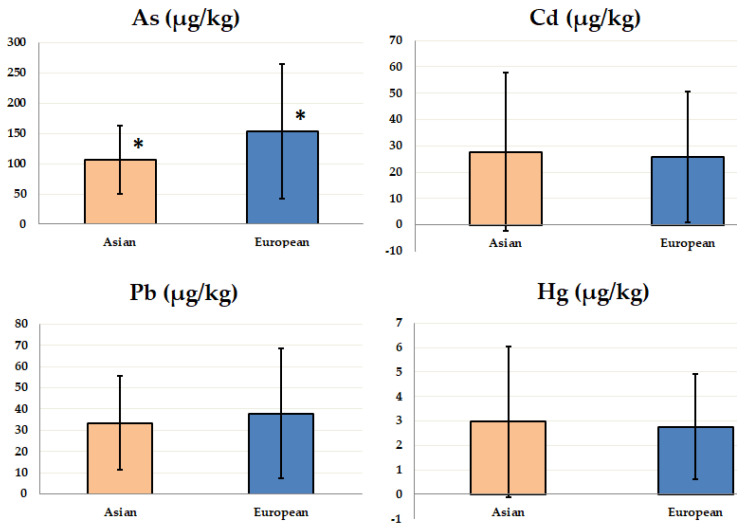
Content of the studied elements (mean ± standard deviation) in samples from Asian and European markets. * *p* < 0.05.

Taking into account the country of origin of the studied products, the analyses were carried out on 53 items produced in Asia and 27 samples from the European markets. The average As content in European rice (153.4 ± 110.9 µg/kg) was higher than in Asian rice (106.4 ± 56.8 µg/kg) and this difference was statistically significant (*p* < 0.05). However, in the remaining analyses no significant differences were found. The following mean contents of the studied elements in Asian and European samples were detected: Cd 27.7 ± 30.1 µg/kg vs. 25.7 ± 24.8 µg/kg, Pb 33.5 ± 22.2 µg/kg vs. 38.0 ± 30.7 µg/kg, and Hg 3.0 ± 3.1 µg/kg vs. 2.8 ± 2.2 µg/kg. The determined contents of the studied elements with regard to the market of origin are presented in Figure 1.

### 3.2. Health Risk Assessment for Polish Population

The health risk indicators for each product individually, as well as considering all separated subgroups, were calculated based on the equations described in the section on materials and methods. Table 6 shows the values of EDI and EWI (EDI value multiplied by 7) indicators for the Polish population. The overall mean EDI of As was established as 0.00062 mg/day. Among the subgroups, red rice samples had the highest average EDI of As (0.00126 mg/day), whereas pasta samples had the lowest EDI score (0.00027 mg/day). The minimum EDI was established for black rice (0.00002 mg/day), whereas the maximum value (0.00281 mg/day) was for red rice. Regarding all the products tested, the EDI of Cd was calculated at 0.00013 mg/day. Flour samples proved to contain the highest EDI of Cd (0.00025 mg/day), whereas basmati rice products contained the lowest (0.00007 mg/day). The EDI of Cd for an individual product ranged from an undetectable level for flakes to 0.00086 mg/day for parboiled rice. As for the next studied element, the average EDI of the Pb value for all the products tested was established as 0.00019 mg/day. Taking into account the subdivision into groups, red rice was found to have the minimum average EDI of Pb (0.00010 mg/day), whereas expanded rice had the maximum level (0.00040 mg/day). The individual product with the greatest EDI of Pb was expanded rice (0.0068 mg/day), in contrast to flakes, where the EDI was nearly zero. The lowest average EDI of Hg for all the products (0.00001 mg/day) was estimated. In 10 out of all the 12 subgroups of rice products, the EDI of Hg had the lowest value (0.00001 mg/day). In the remaining two subgroups (pasta and parboiled rice), the EDI was calculated to be 0.00002 mg/day. The minimum value of the EDI of Hg, which was not detectable, was recorded in one half of the subgroups of products. The greatest EDI was observed in the parboiled rice sample (0.00008 mg/day). 

Although the acceptable standards (set by the PNFSS for As and by the EC for Cd, Pb, and Hg), were found to have been exceeded in individual samples based on the calculated THQ for the Polish population, no increased risk resulting from the intake of the examined toxic elements with rice was found (the reference value was <1). The assessed THQ, HI, and CR are summarized in Table 7. However, the greatest average THQ for As, estimated considering all the products tested (0.0294), varied from 0.0130 for pasta to 0.0601 for the red rice samples. The THQ for As reached the maximum in the case of red rice (0.1339), whereas black rice had the minimum level (0.0010). The mean THQ for Cd for all the samples was 0.0018, whereas in the subgroups, flour had the highest value (0.0036) and basmati rice the lowest (0.0001). Parboiled rice was the product with the greatest THQ for Cd (0.0123), in contrast to flakes, where the THQ was at an undetectable level. The overall THQ for Pb was similar to the THQ for Hg (0.0008 and 0.0007, respectively). Expanded rice was the subgroup characterized by the highest THQ for Pb (0.0016), whereas red rice and flakes had the lowest THQ (0.0004). Analyzing the THQ for Pb in individual products, an undetectable value was observed in the flakes sample, whereas the expanded rice product had the highest content (0.0028). Taking into account the division into subgroups, the flour subgroup had the lowest THQ for Hg (0.0004) and pasta had the highest (0.0009). Considering the individual products, the THQ for Hg ranged from undetectable for white rice to 0.0123 for parboiled rice.

HI was used to calculate the total exposure to the intake of the studied toxic elements found in rice products over a lifetime. In our investigation, it was estimated at 0.0327, which, similarly to THQ, poses no increased health risk (the reference value was <4). However, the summary of exposure to the studied elements reached the maximum value for red rice (0.0624) and the minimum for basmati rice (0.0158). Separately analyzed, HI proved the lowest in the white rice sample (0.0120) and the highest in red rice (0.1349).

The CR for elements with proven pro-cancerogenic effects was estimated based on our research as well as the available statistics concerning average rice consumption in the Polish population. Our results indicate that the risk of cancer due to the consumption of the researched products is low.

### 3.3. Health Risk Assessment for Thai Population

In the case of the Thai population, the health risk assessment was estimated based on the results of the 18 products that had been imported from Thailand. The measured content as well as the values of the indicators calculated for this population are summarized in Table 8. The following average concentrations of the studied elements were determined: As 98.73 ± 55.20 µg/kg, Cd 40.96 ± 42.18 µg/kg, Pb 31.41 ± 23.22 µg/kg, and Hg 2.74 ± 1.93 µg/kg. Based on the obtained results, the highest EDI was estimated for As (0.530 mg/day), followed by the EDI of Cd (0.220 mg/day), Pb (0.169 mg/day), and Hg (0.015 mg/day). Our results indicate that the average rice consumption in the Thai population may be related to adverse health effects associated with As intake. This may be due to the fact that the THQ for As in our analysis was greater than one (1.766). As far as the other elements are concerned, the estimated THQ value was lower and did not exceed the established threshold (THQ for Cd, Pb, and Hg: 0.220; 0.048, and 0.049, respectively). However, the HI was estimated at 2.083, which indicates that the additive effect of the intake of the toxic elements with rice could cause adverse health outcomes. The CR for the Thai population was established for As as 0.0000477 and for Cd as 0.0000831. These values may suggest a potential weak cancerogenic influence of these elements. The lowest CR was established for Pb intake (0.0000001).

### 3.4. Mean BMDL, PTMI, and PTWI Values for Polish and Thai Populations

As regards BMDL, PTWI, and PTMI for both the Polish and Thai populations, no exceedance of the defined limits was found. All obtained results are presented in Table 9. However, the values of the assessed indicators were nearly 60 times higher for As, 72 times higher for Hg, and about 120 times higher for Cd and Pb in the Thai than in the Polish population. The calculated BMDL indicator of As for the Thai population (0.5297 µg/kg BW/day) did not exceed the maximum reference limit (3 µg/kg BW/day). On the other hand, the value of this indicator for the Polish population (0.0088 µg/kg BW/day) was about 340 times lower than the established reference limit. In addition, the value of BMDL of Pb for the Thai population slightly exceeded the lower limit (0.2195 µg/kg BW/day), whereas for the Polish population, it was estimated at 0.0018 µg/kg BW/day. The PTMI of Cd and the PTWI of Hg in the Polish population were estimated at 0.0551 µg/kg BW/month and 0.0014 µg/kg BW/week, respectively, and in the Thai population, at 6.5849 µg/kg BW/month and 0.1014 µg/kg BW/week, respectively. Based on the results obtained in the study and concerning the reference limits established for each of the elements (Table 9), we also estimated the level of rice consumption that could be associated with adverse health effects from exposure to toxic elements. The following results were obtained: As—over 1700 g/day, Cd—more than 2200 g/month, Pb for the lower BMDL limit—55 g/day and for the higher—over 8000 g/day, and (the greatest safe limit) for Hg—over 14,000 g/week.

## 4. Discussion

In the study, we included all the 12 types of rice products available on the Polish market. Assessment of heavy metal contamination is one of the key elements taken into account in evaluation of food quality and safety. In our research, the average contents of toxic elements in all the studied products amounted to As 123.5 ± 77.1 µg/kg, Cd 25.7 ± 26.5 µg/kg, Pb 37.5 ± 29.3 µg/kg, and Hg 2.8 ± 2.6 µg/kg. The results obtained by other authors regarding the measured contents are summarized in Table 10 and those concerning the assessment of the health risk indicators in Table 11.

Results comparable to ours (Table 4) can be found in a study by Sommella et al. [25].

Other Italian researchers evaluated the mean concentration of Cd in products available on the local markets, such as white rice, in which the amount of the metal was 92 µg/kg, and brown rice, in which it was 62 µg/kg. These results were nearly three to four times higher compared to those obtained in this work (Table 4). However, the authors studied a larger number of samples of white and brown rice. Based on the obtained results, the authors estimated the EWI for the Italian population at 0.104 mg/week for brown rice and 0.065 mg/week for white rice [26]. Those values were higher than the ones calculated for the Polish population in our analysis, probably due to greater rice consumption as well as the higher concentrations measured.

In Skendi et al. [27], similar to the results obtained in our study, no product was found to exceed the European standards, established for Cd as 200 µg/kg. The highest Cd concentration (147 µg/kg) was detected in imported white rice. In our analyses (Table 4), the maximum Cd level was higher (171.6 µg/kg), and the median was nearly three times lower (17.9 µg/kg). The amount of Pb exceeded the allowed limit in one sample of imported basmati rice (215 µg/kg) [27]. No exceedance of the standards regarding Pb concentration was demonstrated in our research and the median Pb (24.5 µg/kg) was two times lower. However, the content of Pb was related to the content of Cd in the white and basmati rice samples (r = 0.78, *p* < 0.05).

In another European study by Pinto et al. [28], the average content of As, Cd, and Pb in white (170 ± 6 µg/kg, 11 ± 10 µg/kg, 3 ± 2 µg/kg), parboiled (160 ± 50 µg/kg, 5 ± 3 µg/kg, 3 ± 2 µg/kg), brown (210 ± 50 µg/kg, 10 ± 7 µg/kg, 5 ± 4 µg/kg), and wild (180 ± 30 µg/kg, 9 ± 6 µg/kg, 2 ± 2 µg/kg) rice were determined. When comparing those results with our work (Table 4), As concentration in our samples was lower in each subgroup, but Cd and Pb levels were from four to 10 times higher. To estimate the health risk of consuming the studied products by the Iberian population (residents of both Portugal and Spain), the authors calculated the value of PTWI. The results were presented as the percentage of the referenced PTWI value (7 µg/kg for Cd and 25 µg/kg for Pb). It should be noted that in 2011, the JECFA withdrew the previously established PTWI for Pb [17], which was taken into account in Pinto et al.’s investigation. Nevertheless, the studied products could be regarded as safe to consume for this population [28].

In a study by Brombach et al. [29], the average Hg content in all samples (basmati, white, wild, risotto and others, flour, noodles, pre-cooked milled baby rice, and cakes for toddlers) was 3.04 ± 2.7 µg/kg, ranging from 0.53 µg/kg to 11.1 µg/kg, whereas the mean MeHg concentration was 1.91 ± 1.07 µg/kg (0.11–6.45 µg/kg). Importantly, the percentage of MeHg in the total Hg content in the studied material was determined to be 71 ± 26%, although the statistical analyses did not show any significant differences. When the country of origin was taken into account, there were no differences in Hg or MeHg content between samples from European cultivations compared to rice produced in China, Taiwan, Thailand, and the USA. The results obtained in our work were similar (Table 4, except for MeHg, because speciation was not performed). We also did not notice any differences in Hg content with regard to the country of origin.

The latest study by Menon et al. [30] showed that As content in organically and non-organically cultivated rice differed statistically. The researchers observed this difference in the samples of white (*p* < 0.001) and brown rice (*p* < 0.05). The authors performed a speciation of iAs compounds in 42 of the studied samples and observed that iAs constituted 73% (36–95%) of the total sum of all the As species and these results were similar to those obtained by Liao et al. [31], where iAs(III) content was 53.26–83.03% and iAs(V) 3.45–8.40%. The results of As content were comparable to those identified in our research (Table 4); however, we could not analyze our material with regard to the type of cultivation (organic or non-organic) due to insufficient information. The EDI values of iAs exposure for males and females were 0.0019 mg/day and 0.0020 mg/day, respectively. Furthermore, the THQ for iAs was found to be 0.09 for females and slightly lower (0.8) for males [30]. In this study, the EDI for As was estimated at 0.00062 mg/day, which was higher than in Menon et al. [30]. However, we did not measure iAs content and there is a lack of information about the average rice consumption in Poland according to gender.

A summary of Iranian studies conducted between 2011 and 2018 assessed the content of As in rice samples available on that market (both produced and imported). It demonstrated that As content ranged from 9 µg/kg to 2700 µg/kg. Imported grains had higher As levels than domestic ones. The highest observed value was nearly five times higher than the greatest As content detected in our samples (562.2 µg/kg). The EWI of As for that population was estimated at 0.0063 mg/week [36]. A health risk assessment by indicating the THQ, total THQ (HI in our study), and CR for the adult Iranian population based on the measured toxic element content in rinsed or traditionally cooked grains was performed by Shariatifar et al. [38]. The health risk indicators for the Iranian population compared with the Polish indicators were higher as a result of both the determined concentrations and considerably higher consumption.

In the latest large Chinese investigation, among all the analyzed samples, only three exceeded the Chinese National Food Safety Standard (20 µg/kg). When the dietary exposure of Hg via rice consumption was evaluated, the PTWI indicator was assessed as 0.0014–0.0017 µg/kg/week. However, Hg exposure in populations with the highest rice consumption compared to the lowest was nearly 7 times higher. The higher exposure was also determined in more polluted areas. The authors compared the obtained results with previous investigations and observed that in the years 2007–2017, the decline in the average locally produced rice ranged from 17.7% to 42.5% [37]. It must be noted that the Chinese standards indicate higher limit values for Hg in rice compared to European requirements (20 vs. 10 µg/kg). Therefore, when comparing to the European guidelines, the number of samples exceeding the limits would probably be higher. The average Hg concentration in our results (Table 4) was lower than in Xu et al. [37].

The content of heavy metals and the health risk assessment among four locally cultivated rice species (labelled as A, B, C, D, *n* = 55) in Thailand was analyzed by Kukusamude et al. [32]. Significant differences between As and Cd content among the studied species were observed (*p* < 0.001 and *p* = 0.001). When Thai rice was analyzed in our study, the average As content was 98.7 µg/kg (4.1–206.0 µg/kg) and Cd 41 µg/kg (8.0–171.6 µg/kg). In our investigation, lower As content but higher Cd levels were determined. The EWI of As for Thai females and males ranged from 0.046 mg/week and 0.045 mg/week (for variety A) to 0.381 mg/week and 0.380 mg/week (for variety D), respectively. When taking into account the EWI of Cd, the lowest values for females and males, respectively, were estimated for variety A as 0.010 mg/week and 0.009 mg/week and the highest for variety B as 0.0478 mg/week and 0.0475 mg/week. The THQ values of As/Cd amounted to A 0.425/0.027, B 2.79/0.131, C 1.65/0.035, and D 3.58/0.057 [32]. On the other hand, when the EWI and THQ for the Thai population was estimated in our research, the values were higher.

The contents of toxic as well as non-toxic elements in rice samples cultivated in Argentina (Latin America) were investigated by Londonio et al. [33]. As content was higher than that identified in our investigation (Table 4), whereas the concentrations of other toxic elements were lower. The EWI for the Argentinian population was also estimated. The average rice consumption per capita/year in this country was 15 kg. The highest EWI was calculated for As (0.247 mg/week), followed by Pb (0.0403 mg/week), Cd (0.00715 mg/week), and the lowest for Hg (<0.02 mg/week) [33]. The values of EWI for the Polish population were about 20 times lower.

Canadian researchers conducted a study assessing Hg content in 89 different rice varieties imported from eight countries: USA (48%), Thailand (17%), Pakistan (13%), India (8%), Italy (4%), Vietnam (3%), Argentina (2%), China (2%), and Spain (2%). No significant differences between brown and white rice were observed. However, the content of Hg in black and red as well as long grain rice was statistically higher than in short/medium and basmati rice (*p* < 0.05). The results were comparable to those obtained in our study (Table 4), however, as regards Hg content, we observed no significant differences between the rice subgroups (Table 5). The EDI for the Canadian population was estimated at 0.0000067 mg/day. When the authors considered extreme consumption, the weekly exposure would reach 0.037 mg/kg/week [34]. Both the Hg content measured and the EDI calculated were similar to our findings.

Gluten-free and gluten-containing products available on the USA market were analyzed by Punshon and Jackson [35]. We did not incorporate enriched white rice due to its unavailability on our market, but the results by Punshon and Jackson were comparable to our observations (Table 4).

An extensive study assessing Cd content in rice produced by 12 countries was conducted by Meharg et al. [39]. The samples were collected from seven countries in Asia (Bangladesh, Cambodia, Ghana, India, Japan, Nepal, Sri Lanka, and Thailand), three in Europe (France, Italy, and Spain), one in Africa (Ghana), and one in North America (USA). The highest average concentration was detected in Bangladeshi rice (99 µg/kg), whereas the lowest was in French (10 µg/kg). The minimum Cd contents (below the limit of detection) were observed in samples from Sri Lanka and Bangladesh. However, in another sample of Bangladeshi rice, the greatest content (1310 µg/kg) was also determined. The risk of weekly Cd intake with rice consumption for Cambodian, French, and Ghanaian residents was estimated to be low, but higher for the Spanish, Italian, and US population. For the remaining countries, both higher Cd content and greater consumption of rice per day caused higher exposure to Cd. The results of this investigation revealed that Cd pollution is a major concern worldwide. For countries that do not cultivate rice, the country of origin of imported grains may play an important role in Cd exposure [39]. However, in our research, statistically significant differences were detected as regards the country of origin (Figure 1)—European rice had greater (*p* < 0.05) content of As compared to Asian rice.

There are several reports describing post-harvesting methods that could reduce the content of heavy metals in grains. One of them is grain polishing. Jo and Todorov [4] conducted research into brown rice and compared the concentration of selected elements in the grains and in bran when 30%, 50%, 70%, and 100% of the grain was polished. In unpolished grain the following levels of the studied elements (As, Cd, Hg, and Pb) were detected: 262 µg/kg, 14 µg/kg, 4.8 µg/kg, and 4 µg/kg, respectively. However, in the first step of the polishing (30%), differences in the content of heavy metals in the grains compared to the bran were observed (As 221 vs. 598 µg/kg, Cd 14 vs. 23 µg/kg, Hg 4.1 vs. 8.3 µg/kg, and Pb 4 vs. 17 µg/kg, respectively). After the polishing had been completed, the following concentrations were found: As 205 vs. 599 µg/kg, Cd 12 vs. 22 µg/kg, Hg 3.2 vs. 7.1 µg/kg, and Pb 3 vs. 11 µg/kg in the grains and the bran, respectively. Higher contents of all the elements were detected in the bran than in the endosperm, which indicates that whole rice grain contains greater amounts not only of the essential, but also of toxic elements [4]. In our analysis, significant differences in As, Cd, and Pb content in some whole grain subgroups compared to products from polished grains were observed. In addition, similar observations were reported in Menon et al. [30], Lin et al. [34], and Punshon and Jackson [35].

Another post-harvesting method that could considerably reduce As content in rice involves washing raw rice several times before cooking as well as cooking in large amounts of water. The study of the influence of ordinary and pressure cooking on As content in rice revealed that iAs concentration decreased by 50–36–79.17% and 49.48–77.26%, respectively. The reduction was associated with the release of iAs into cooking water. On the other hand, organic As content remained unchanged [40]. Shariatifar et al. [38] also observed decreased concentration of As, Cd, and Pb in rinsed rice grains in comparison to traditionally cooked rice, although a reduction in essential element content was also noted [38].

Liao et al. [31] observed that the methods of cooking rice, ordinary and pressure, reduced the total Hg content by 5.11–65.41%. In particular, iHg concentration decreased by 60.89% and 41.18%, respectively. However, no reduction in MeHg content after cooking was detected [31]. In their investigation, of the total Hg content, iHg constituted 69.51–80.91% and MeHg 19.09–30.49%.

Al-Saleh and Abduljabbar [41] investigated the influence of two different methods, soaking and rinsing rice grains, on heavy metal content in 61 samples collected in Saudi Arabia. Soaking for 20 min removed 98.2% of As, 86.9% of Cd, 93.0% of MeHg, and 97.8% of Pb, while rinsing three times with deionized water removed 96.6%, 64.9%, 91.6% and 97.0%, respectively. A statistically significant higher content of the three studied elements (Cd, MeHg, and Pb) in rinsed rather than soaked grains was detected. The following average values of EDI of soaked or rinsed rice were established for that population: As 3.769/3.407 µg/kg/week, Cd 0.279/0.503 µg/kg/week, MeHg 0.271/0.309 µg/kg/week, and Pb 0.638/1.068 µg/kg/week. When non-cancerogenic risk was estimated, the hazard quotient (HQ) of the soaked/rinsed grains for the toxic elements (As, Cd, MeHg, Pb) amounted to 1.795/1.622, 0.040/0.072, 0.388/0.441, and 0.026/0.044, respectively. All the samples tested had the HQ of As above one, which indicates a higher risk of negative health effects [41].

In vivo investigations in swine models indicated that the gastrointestinal absorption of iAs from rice consumption ranged from 85 to 90%, while organic As species had significantly lower absorption (20–31%) [42].

An in vitro experiment conducted by Sharafi et al. [43] demonstrated that among the three toxic elements whose content had been studied in three different rice varieties, Cd had the highest total bioaccessibility (28.75%), followed by As and Pb (each 20.65%). Three digestion phases were distinguished: oral, gastric, and small intestinal. The following bioaccessibility rates were observed: As and Pb—2.60%, 15.55%, 2.60%, and Cd—3.30%, 22.65%, and 2.30%, respectively. The authors concluded that the higher gastric bioaccessibility may be related to low pH, which increases the solubility of metals [43]. Another in vitro investigation demonstrated that the gastrointestinal bioaccessibility of Hg from raw rice ranged from 65.73 to 76.10% and was significantly higher than gastric bioaccessibility (50.56–55.61%). However, the levels of As bioaccessibility were higher than the ones obtained by Sharafri et al. [43] and ranged from 60.48% in gastric digestion, reaching the highest value in the gastrointestinal phase—80.32%. The authors observed that all the bioaccessible levels were lower than the measured content [44]. In addition, the investigation by Althobiti et al. [44] demonstrated that the bioaccessibility of As from raw rice varied depending on the country of origin: from 16% in the case of Lebanese rice to 88.5% in the case of Iranian rice. Furthermore, nearly 3% of As was removed by washing in Egyptian rice, while a decrease of over 40% in US rice was detected.

To our knowledge, this study is the first ever to have analyzed so many different rice products including pasta, flakes, flour, waffles, and expanded rice. Moreover, we estimated several health risk indicators associated with exposure to toxic elements from rice products among the Polish population, which had not been assessed previously. In order to reduce possible exposure to toxic elements, both the choice of polished grains and their proper preparation for cooking should be recommended, in particular when consumed in larger amounts. In some population subgroups in Poland, such as people suffering from gluten intolerance (celiac disease, non-celiac disease), the consumption of rice products may be higher compared to the whole Polish population. However, it was not possible to calculate the dietary exposure to the studied elements among these patients due to the lack of available data on the intake levels in these subgroups. Therefore, we see a great need to conduct a large investigation to complete the missing data in this regard. In addition, further research determining MeHg and iAs speciation in the studied material is required.

## 5. Conclusions

In our study, only individual samples exceeded the established standards. The estimated health risk indicators did not show increased risk of exposure to the toxic elements under consideration as a result of rice consumption for the Polish population. However, the available statistics may be underestimated. The estimations of the highest permissible intake indicated that the daily consumption of 55 g of the studied rice corresponded to the lower BMDL limit of Pb. On the other hand, when the health risk indicators for the Thai population were analyzed, the values were approximately 60–120 times higher than for the Polish population. Therefore, increased consumption of the products we examined may be associated with negative effects on the organism due to the content of toxic elements. Currently available studies demonstrate that contamination of rice with heavy metals poses a problem all over the world. In the countries where rice is cultivated, strategies aimed at preventing the accumulation of heavy metals are important, whereas in the countries that import rice, assessment of its safety is crucial.

## Figures and Tables

**Table 1:** Steps and parameters of microwave digestion of rice and rice products in a closed-loop system (Berghof, Speedwave, Eningen, Germany).

Phase	Temperature [°C]	Pressure [atm]	Time [min]	Power [%]
1.	170	20	10	90
2.	190	30	10	90
3.	210	40	10	90
4.	50	40	18	0

**Table 2:** ICP-MS conditions for As, Cd, Pb determination in rice and rice products.

Parameter	Analytical Conditions		
	As	Cd	Pb
Mode	KED	Standard	Standard
Mass (amu)	75	110 111 113 114	206 207 208
Dwell Time per amu (ms)	50	50	50
Integration Time (ms)	1000	1000	1000
Detector Calibration Mode	Dual	Dual	Dual
Replicates	5	5	5

ICP-MS – Inductively Coupled Plasma-Mass Spectrometry, KED—kinetic energy discrimination.

**Table 3:** Results obtained in the quality control process.

Element	Precision (%)	Recovery (%)	Declared Concentration in CRM (µg/kg)
As	3.2%	98.5%	10
Cd	2.4%	99.0%	7
Pb	2.4%	99.6%	52
Hg	2.2%	102.0%	1.5

CRM—certified reference material.

**Table 4:** Content of As, Cd, Pb, and Hg measured in the studied material.

Type of Rice and Rice Product	*n*	As [µg/kg]		Cd [µg/kg]		Pb [µg/kg]		Hg [µg/kg]	
		X ± SD (Min–Max)	Me (Q_1_–Q_3_)	X ±SD (Min–Max)	Me (Q_1_–Q_3_)	X ± SD (Min–Max)	Me (Q_1_-Q_3_)	X ± SD (Min–Max)	Me (Q_1_–Q_3_)
Flakes	12	122.2 ± 58.1 (56.3–246.1)	109.5 (79.2–169.4)	27.9 ± 22.1 (0.3–73.1)	20.0 (15.0–34.6)	18.4 ± 13.3 (0.4–48.7)	13.2 (11.7–21.9)	2.5 ± 1.7 (0.9–7.2)	2.0 (1.4–2.4)
White	11	96.8 ± 36.1 (46.0–168.9)	96.6 (72.4–126.0)	23.4 ± 22.8 (4.2–81.7)	20.8 (5.8–34.1)	26.2 ± 17.7 (9.7–59.7)	18.6 (11.1–43.7)	2.8 ± 2.7 (0.1–8.3)	1.6 (1.0–4.5)
Basmati	10	55.6 ± 10.3 (39.5–71.5)	57.3 (48.1–61.3)	14.2 ± 15.2 (1.9–39.5)	4.8 (2.5–30.9)	40.4 ± 19.1 (17.0–66.0)	44.9 (20.9–52.9)	3.0 ± 4.3 (0.4–14.8)	1.7 (0.5–3.1)
Parboiled	10	96.7 ± 32.7 (53.6–151.3)	97.1 (73.3–115.8)	33.5 ± 49.8 (8.9–171.6)	16.7 (9.2–25.3)	36.9 ± 17.0 (17.6–64.1)	37.2 (21.4–48.0)	4.0 ± 5.0 (0.1–15.8)	1.8 (0.8–5.8)
Brown	10	204.6 ± 75.2 (113.0–314.5)	199.8 (149.0–277.5)	23.8 ± 29.8 (1.9–103.0)	14.1 (8.8–25.7)	34.1 ± 34.5 (9.3–106.7)	18.4 (15.7–35.6)	2.3 ± 1.7 (0.2–5.9)	1.8 (1.3–3.5)
Waffles	9	178.9 ± 33.5 (124.3–232.9)	173.3 (158.2–202.7)	18.7 ± 14.1 (8.6–40.3)	9.7 (9.2–34.1)	56.4 ± 46.2 (14.8–134.8)	27.6 (19.8–96.5)	3.5 ± 1.7 (0.3–6.2)	3.5 (2.8–4.5)
Expanded	8	105.7 ± 28.4 (66.3–160.8)	104.8 (87.0–117.4)	16.6 ± 12.8 (6.3–46.6)	13.9 (9.0–16.9)	79.2 ± 36.8 (21.4–135.6)	79.3 (54.0–105.1)	2.7 ± 1.9 (1.1–6.5)	1.7 (1.4–3.8)
Pasta	7	54.6 ± 6.3 (48.0–65.2)	54.3 (48.4–58.4)	20.5 ± 11.3 (8.0–37.3)	17.9 (12.5–35.0)	31.6 ± 14.6 (18.9–60.1)	24.5 (23.6–42.9)	3.7 ± 2.4 (1.1–7.3)	4.3 (1.2–5.4)
Black	6	127.8 ± 78.8 (4.2–236.3)	124.4 (101.1–176.5)	44.8 ± 40.9 (8.4–122.1)	32.3 (21.5–52.5)	25.3 ± 10.8 (13.7–42.3)	23.7 (15.6–32.6)	2.4 ± 1.6 (1.3–5.3)	1.6 (1.4–2.7)
Flour	6	100.5 ± 44.7 (65.1–187.3)	89.5 (69.3–102.1)	50.2 ± 21.8 (21.4–85.7)	48.1 (38.8–59.2)	46.3 ± 7.5 (36.5–54.3)	46.6 (39.2–54.3)	1.8 ± 0.8 (1.0–3.1)	1.6 (1.4–2.4)
Red	5	252.2 ± 173.6 (157.5–562.2)	176.6 (176.4–188.6)	18.6 ± 16.3 (2.3–45.1)	14.2 (10.1–21.1)	19.6 ± 11.3 (11.6–39.4)	14.7 (13.7–18.4)	2.7 ± 0.6 (2.1–3.7)	2.5 (2.4–3.0)
Wild	5	132.3 ± 67.4 (14.7–182.7)	157.0 (142.1–165.1)	26.6 ± 16.7 (10.0–54.1)	21.3 (19.7–27.7)	39.1 ± 41.7 (9.6–106.6)	14.6 (12.0–52.9)	2.2 ± 1.5 (1.2–4.8)	1.5 (1.4–2.0)
TOTAL	99	123.5 ± 77.1 (4.2–562.2)	107.2 (66.0–165.1)	25.7 ± 26.5 (0.3–171.6)	17.9 (9.2–35.0)	37.5 ± 29.3 (0.4–135.6)	24.5 (16.2–52.7)	2.8 ± 2.6 (0.1–15.8)	2.1 (1.3–3.6)

X—mean, SD—standard deviation, Min—minimum, Max—maximum, Me—median, Q_1_—lower quartile, Q_3_—upper quartile.

**Table 5:** Significant differences detected in the statistical analysis.

Analyzed Parameter	As	*p*-Value	Cd	*p*-Value	Pb	*p*-Value
Subgroups among which differences were found	pasta—brown rice	*p* < 0.05	Flour—basmati rice	*p* < 0.05	white rice—expanded rice	*p* < 0.05
	pasta—red rice	*p* < 0.05				
	basmati rice—red rice	*p* < 0.05			white rice—red rice	*p* < 0.05
	basmati rice—brown rice	*p* < 0.001				
	waffles—pasta	*p* < 0.001			flakes—expanded rice	*p* < 0.01

*p*-Value—statistical significance level.

**Table 6:** The values of EDI and EWI estimated for the Polish population for each subgroup of studied products.

Type of Rice Product	*n*	EDI [mg/day]/EWI [mg/week]							
		As		Cd		Pb		Hg	
Flakes	12	0.00061 ± 0.00029 (0.00028–0.00123)	0.00428 ± 0.00203 (0.00197–0.00861)	0.00014 ± 0.00011 (0.00000–0.00037)	0.00098 ± 0.00077 (0.00001–0.00256)	0.00014 ± 0.00011 (0.00000–0.00037)	0.00064 ± 0.00046 (0.00001–0.00171)	0.00001 ± 0.00001 (0.00000–0.00004)	0.00008 ± 0.00006 (0.00003–0.00025)
White	11	0.00048 ± 0.00018 (0.00023–0.00084)	0.00339 ± 0.00126 (0.00161–0.00591)	0.00012 ± 0.00011 (0.00002–0.00041)	0.00082 ± 0.00080 (0.00015–0.00286)	0.00013 ± 0.00009 (0.00005–0.00030)	0.00092 ± 0.00062 (0.00034–0.00209)	0.00001 ± 0.00001 (0.00000–0.00004)	0.00010 ± 0.00009 (0.00000–0.00029)
Basmati	10	0.00028 ± 0.00005 (0.00020–0.00036)	0.00194 ± 0.00036 (0.00138–0.00250)	0.00007 ± 0.00008 (0.00001–0.00020)	0.00050 ± 0.00053 (0.00007–0.00138)	0.00020 ± 0.00010 (0.00009–0.00033)	0.00141 ± 0.00067 (0.00060–0.00231)	0.00001 ± 0.00002 (0.00000–0.00007)	0.00010 ± 0.00015 (0.00001–0.00052)
Parboiled	10	0.00048 ± 0.00016 (0.00027–0.00076)	0.00339 ± 0.00114 (0.00188–0.00530)	0.00017 ± 0.00025 (0.00004–0.00086)	0.00117 ± 0.00174 (0.00031–0.00601)	0.00018 ± 0.00009 (0.00009–0.00032)	0.00129 ± 0.00060 (0.00061–0.00224)	0.00002 ± 0.00002 (0.00000–0.00008)	0.00014 ± 0.00017 (0.00000–0.00055)
Brown	10	0.00102 ± 0.00038 (0.00056–0.00157)	0.00716 ± 0.00263 (0.00395–0.01101)	0.00012 ± 0.00015 (0.00001–0.00052)	0.00083 ± 0.00104 (0.00007–0.00361)	0.00017 ± 0.00017 (0.00005–0.00053)	0.00119 ± 0.00121 (0.00033–0.00373)	0.00001 ± 0.00001 (0.00000–0.00003)	0.00001 ± 0.00001 (0.00000–0.00003)
Waffles	9	0.00089 ± 0.00017 (0.00062–0.00116)	0.00626 ± 0.00117 (0.00435–0.00815)	0.00009 ± 0.00007 (0.00004–0.00020)	0.00065 ± 0.00049 (0.00030–0.00141)	0.00028 ± 0.00023 (0.00007–0.00067)	0.00197 ± 0.00162 (0.00052–0.00472)	0.00002 ± 0.00001 (0.00000–0.00003)	0.00012 ± 0.00006 (0.00001–0.00022)
Expanded	8	0.00053 ± 0.00014 (0.00033–0.00080)	0.00370 ± 0.00100 (0.00232–0.00563)	0.00008 ± 0.00006 (0.00003–0.00023)	0.00058 ± 0.00045 (0.00022–0.00163)	0.00040 ± 0.00018 (0.00011–0.00068)	0.00277 ± 0.00129 (0.00075–0.00475)	0.00001 ± 0.00001 (0.00001–0.00003)	0.00009 ± 0.00007 (0.00004–0.00023)
Pasta	7	0.00027 ± 0.00003 (0.00024–0.00033)	0.00191 ± 0.00022 (0.00168–0.00228)	0.00010 ± 0.00006 (0.00004–0.00019)	0.00072 ± 0.00040 (0.00028–0.00131)	0.00016 ± 0.00007 (0.00009–0.00030)	0.00111 ± 0.00051 (0.00066–0.00210)	0.00002 ± 0.00001 (0.00001–0.00004)	0.00013 ± 0.00008 (0.00004–0.00025)
Black	6	0.00064 ± 0.00039 (0.00002–0.00118)	0.00447 ± 0.00276 (0.00015–0.00827)	0.00022 ± 0.00020 (0.00004–0.00061)	0.00157 ± 0.00143 (0.00029–0.00427)	0.00013 ± 0.00005 (0.00007–0.00021)	0.00088 ± 0.00038 (0.00048–0.00148)	0.00001 ± 0.00001 (0.00001–0.00003)	0.00008 ± 0.00005 (0.00004–0.00019)
Flour	6	0.00050 ± 0.00022 (0.00033–0.00094)	0.00352 ± 0.00157 (0.00228–0.00655)	0.00025 ± 0.00011 (0.00011–0.00043)	0.00176 ± 0.00076 (0.00075–0.00300)	0.00023 ± 0.00004 (0.00018–0.00027)	0.00162 ± 0.00026 (0.00128–0.00190)	0.00001 ± 0.00000 (0.00001–0.00002)	0.00006 ± 0.00003 (0.00004–0.00011)
Red	5	0.00126 ± 0.00087 (0.00079–0.00281)	0.00883 ± 0.00608 (0.00551–0.01968)	0.00009 ± 0.00008 (0.00001–0.00023)	0.00065 ± 0.00057 (0.00008–0.00158)	0.00010 ± 0.00006 (0.00006–0.00020)	0.00068 ± 0.00040 (0.00040–0.00138)	0.00001 ± 0.00000 (0.00001–0.00002)	0.00010 ± 0.00002 (0.00007–0.00013)
Wild	5	0.00066 ± 0.00034 (0.00007–0.00091)	0.00463 ± 0.00236 (0.00051–0.00640)	0.00013 ± 0.00008 (0.00005–0.00027)	0.00093 ± 0.00058 (0.00035–0.00189)	0.00020 ± 0.00021 (0.00005–0.00053)	0.00137 ± 0.00146 (0.00034–0.00373)	0.00001 ± 0.00001 (0.00001–0.00002)	0.00008 ± 0.00005 (0.00004–0.00017)
TOTAL	99	0.00062 ± 0.00039	0.00865 ± 0.00540	0.00013 ± 0.00013	0.00180 ± 0.00185	0.00019 ± 0.00015	0.00262 ± 0.00205	0.00001 ± 0.00001	0.00020 ± 0.00018

EDI – estimated daily intake, EWI – estimated weekly intake (EWI).

**Table 7:** THQ, HI, and CR estimated for Polish population.

Type of Rice Product	*n*	THQ X ± SD (Min–Max)				HI X ± SD (Min–Max)
		As	Cd	Pb	Hg	
Flakes	12	0.0291 ± 0.0138 (0.0134–0.0586)	0.0020 ± 0.0016 (0.0000–0.0052)	0.0004 ± 0.0003 (0.0000–0.0010)	0.0006 ± 0.0004 (0.0002–0.0017)	0.0320 ± 0.0132 (0.0168–0.0607)
White	11	0.0230 ± 0.0086 (0.0110–0.0402)	0.0017 ± 0.0016 (0.0003–0.0058)	0.0005 ± 0.0004 (0.0002–0.0012)	0.0007 ± 0.0006 (0.0000–0.0020)	0.0259 ± 0.0079 (0.0120–0.0408)
Basmati	10	0.0132 ± 0.0025 (0.0094–0.0170)	0.0010 ± 0.0011 (0.0001–0.0028)	0.0008 ± 0.0004 (0.0003–0.0013)	0.0007 ± 0.0010 (0.0001–0.0035)	0.0158 ± 0.0024 (0.0129–0.0212)
Parboiled	10	0.0230 ± 0.0078 (0.0128–0.0360)	0.0024 ± 0.0036 (0.0006–0.0123)	0.0008 ± 0.0003 (0.0004–0.0013)	0.0024 ± 0.0036 (0.0006–0.0123)	0.0271 ± 0.0103 (0.0144–0.0496)
Brown	10	0.0487 ± 0.0179 (0.0269–0.0749)	0.0017 ± 0.0021 (0.0001–0.0074)	0.0007 ± 0.0007 (0.0002–0.0022)	0.0006 ± 0.0004 (0.0001–0.0014)	0.0517 ± 0.0173 (0.0287–0.0769)
Waffles	9	0.0426 ± 0.0080 (0.0296–0.0554)	0.0013 ± 0.0010 (0.0006–0.0029)	0.0012 ± 0.0009 (0.0003–0.0028)	0.0008 ± 0.0004 (0.0001–0.0015)	0.0459 ± 0.0086 (0.0334–0.0611)
Expanded	8	0.0252 ± 0.0068 (0.0158–0.0383)	0.0012 ± 0.0009 (0.0005–0.0033)	0.0016 ± 0.0008 (0.0004–0.0028)	0.0006 ± 0.0004 (0.0003–0.0015)	0.0286 ± 0.0065 (0.0214–0.0408)
Pasta	7	0.0130 ± 0.0015 (0.0114–0.0155)	0.0015 ± 0.0008 (0.0006–0.0027)	0.0006 ± 0.0003 (0.0004–0.0012)	0.0009 ± 0.0006 (0.0003–0.0017)	0.0160 ± 0.0017 (0.0141–0.0187)
Black	6	0.0304 ± 0.0188 (0.0010–0.0563)	0.0032 ± 0.0029 (0.0006–0.0087)	0.0005 ± 0.0002 (0.0003–0.0009)	0.0006 ± 0.0004 (0.0003–0.0013)	0.0347 ± 0.0163 (0.0104–0.0577)
Flour	6	0.0239 ± 0.0106 (0.0155–0.0446)	0.0036 ± 0.0016 (0.0015–0.0061)	0.0009 ± 0.0002 (0.0007–0.0011)	0.0004 ± 0.0002 (0.0002–0.0007)	0.0289 ± 0.0095 (0.0207–0.0473)
Red	5	0.0601 ± 0.0413 (0.0375–0.1339)	0.0013 ± 0.0012 (0.0002–0.0032)	0.0004 ± 0.0002 (0.0002–0.0008)	0.0007 ± 0.0001 (0.0005–0.0009)	0.0624 ± 0.0406 (0.0393–0.1349)
Wild	5	0.0315 ± 0.0160 (0.0035–0.0435)	0.0019 ± 0.0012 (0.0007–0.0039)	0.0008 ± 0.0009 (0.0002–0.0022)	0.0005 ± 0.0004 (0.0003–0.0011)	0.0347 ± 0.0157 (0.0077–0.0469)
TOTAL	99	0.0294 ± 0.0184	0.0018 ± 0.0019	0.0008 ± 0.0006	0.0007 ± 0.0006	0.0327 ± 0.0181
Mean CR for all	99	0.0000009	0.0000008	0.0000000	N/A	N/A

THQ—target hazard quotient, HI—hazard index, CR—cancer risk, X—mean, SD—standard deviation, Min—minimum, Max—maximum, N/A—not applicable.

**Table 8:** Content of As, Cd, Pb, and Hg in rice samples imported from Thailand (*n* = 18) and the health risk indicators calculated for the Thai population.

Element	As	Cd	Pb	Hg
Measured content [µg/kg] X ± SD (Min–Max)	98.73 ± 55.20 (4.21–205.95)	40.96 ± 42.18 (8.03–171.64)	31.41 ± 23.22 (9.58–106.65)	2.74 ± 1.93 (0.77–7.26)
EDI [mg/day] X ± SD (Min–Max)	0.530 ± 0.296 (0.023–1.105)	0.220 ± 0.226 (0.043–0.921)	0.169 ± 0.125 (0.051–0.572)	0.015 ± 0.010 (0.004–0.039)
EWI [mg/week] X ± SD (Min–Max)	3.709 ± 2.074 (0.158–7.737)	1.539 ± 1.584 (0.302–6.448)	1.180 ± 0.872 (0.360–4.007)	0.103 ± 0.072 (0.029–0.273)
THQ X ± SD (Min–Max)	1.766 ± 0.987 (0.075–3.684)	0.220 ± 0.226 (0.043–0.921)	0.048 ± 0.036 (0.015–0.164)	0.049 ± 0.034 (0.014–0.130)
HI X ± SD (Min–Max)	2.083 ± 1.021 (0.782–4.019)			
mean CR	0.0000477	0.0000831	0.0000001	N/A

X—mean, SD—standard deviation, Min—minimum, Max—maximum, EDI—estimated daily intake, EWI—estimated weekly intake, THQ—target hazard quotient, HI—hazard index, CR—cancer risk, N/A—not applicable.

**Table 9:** BMDL, PTMI, and PTWI values of studied elements for Polish and Thai populations.

Population	Element	As BMDL [µg/kg BW/Day]	Cd PTMI [µg/kg BW/Month]	Pb BMDL [µg/kg BW/Day]	Hg PTWI [µg/kg BW/Week]
Polish		0.0088	0.0551	0.0018	0.0014
Thai		0.5297	6.5849	0.2195	0.1014
Reference limit		3.00	25.00	0.02 - 3.00	4.00

BMDL—benchmark dose lower confidence limit, PTMI—provisional tolerable monthly intake, PTWI—provisional tolerable weekly intake.

**Table 10:** Content of As, Cd, Pb, and Hg in rice determined by other authors.

References	Place of Sample Collection	Study Material	Element (µg/kg)			
			As	Cd	Pb	Hg
Sommella et al. [25]	Italy	Rice (without subdivision into types, *n* = 101)	Min–Max: 70–470	Min–Max: 0–160	-	-
Pastorelli et al. [26]	Italy	White rice (*n* = 41)	X = 92	-	-	-
		Brown rice (*n* = 34)	X = 62	-	-	-
Skendi et al. [27]	Greece	Rice (without subdivision into types, *n* = 26)	-	Me = 60	Me = 50	-
Pinto et al. [28]	Portugal and Spain	White rice (*n* = 56)	X ± SD = 170 ± 6	X ± SD = 11 ± 10	X ± SD = 3 ± 2	-
		Parboiled rice (*n* = 13)	X ± SD = 160 ± 50	X ± SD = 5 ± 3	X ± SD = 3 ± 2	-
		Brown rice (*n* = 11)	X ± SD = 210 ± 50	X ± SD = 10 ± 7	X ± SD = 5 ± 4	-
		Wild rice (*n* = 6)	X ± SD = 180 ± 30	X ± SD = 9 ± 6	X ± SD = 2 ± 2	-
Brombach et al. [29]	United Kingdom, Germany, and Switzerland	All studied rice products (*n* = 87)	-	-	-	X ± SD = 3.04 ± 2.7
		Wild rice (*n* = 3)	-	-	-	Min–Max: 1.63–5.83
		Basmati rice (*n* = 8)	-	-	-	Min–Max: 1.24–6.56
		White rice (*n* = 19)	-	-	-	Min–Max: 0.53–11.14
Menon et al. [30]	United Kingdom	All studied rice products (*n* = 55)	X = 150 (Min–Max: 10–370)	-	-	-
Xu et al. [31]	China	Rice (without subdivision into types, *n* = 709)	-	-	-	X = 4.03
						(Min–Max: 0.638–31.7)
Kukusamude et al. [32]	Thailand	Locally cultivated rice (*n* = 55)				
		Type A (*n* = 5)	X = 110 (Min–Max: 81–135)	X = 24 (Min–Max: 3–92)	-	-
		Type B (*n* = 15)	X = 160 (Min–Max: 67–254)	X = 21 (Min–Max: 6–55)	-	-
		Type C (*n* = 9)	X = 190 (Min–Max: 77–283)	X = 6 (Min–Max: 2–10)	-	-
		Type D (*n* = 26)	X = 240 (Min–Max: 166–402)	X = 9 (Min–Max: 3–29)	-	-
Londonio et al. [33]	Argentina	All studied rice products (*n* = 24)	X = 237	X = 6	X = 25	Below the limit of detection
			(Min–Max: 67–858)	(Min–Max: 0–24)	(Min–Max: 0–139)	
Lin et al. [34]	Canada	Basmati rice (*n* = 23)	-	-	-	X = 1.7
		Black and red (*n* = 7)	-	-	-	X = 4.7
		Jasmine (*n* = 12)	-	-	-	X = 2.9
		Long grain (*n* = 22)	-	-	-	X = 4.1
		Short and medium grain (*n* = 25)	-	-	-	X = 2.0
		White rice (*n* = 67)	-	-	-	X = 2.6
Punshon and Jackson [35]	United States of America	All studied products (*n* = 67), of which:				
		Rice flour (*n* = 4)	X = 112	X = 26	X = 4	X = 2.2
		White rice (*n* = 5)	X = 94	X = 38	X = 19	X = 1.4
		Brown rice (*n* = 6)	X = 183	X = 27	X = 8	X = 1.8
		Enriched white (*n* = 7)	X = 177	X = 10.9	X = 21	X = 1.9

X—Mean, SD—standard deviation, Me—median, Min—minimum, Max—maximum.

**Table 11:** Values of health risk indicators estimated in our research and by other authors.

Type of Health Risk Indicator	Element	Product/Group of Products	Our Results	Other Authors	Population	References
EDI [mg/day]	Hg	All rice products	0.00001	0.0000067	Canadian	Lin et al. [34]
EDI [mg/day]	iAs	All rice products	0.00062 *	0.0019–0.0020	British	Menon et al. [30]
EWI [mg/week]	Cd	Brown rice	0.00083	0.104	Italian	Pastorelli et al. [26]
		White rice	0.00082	0.065		Pastorelli et al. [26]
EWI [mg/week]	As/Cd	Rice A (white) Rice B (brown) Rice C (white) Rice D (white)	0.00083 (brown) 0.00082 (white)	0.045–0.046/0.01 0.300–0.301/0.013–0.014 0.173–0.174/0.048 0.380/0.020	Thai	Kukusamude et al. [32]
EWI [mg/week]	As	All rice products	0.00865	0.0063	Iranian	Hashempour-Baltork et al. [36]
EWI [mg/week]	As/Cd/Pb/Hg	All rice products	0.00865/0.00180/0.00262/0.00020	0.247/0.00715/0.0403/<0.02	Argentinian	Londonio et al. [33]
PTWI [% PTWI]	Cd/Pb	Brown	0.17/0.07	0.50/0.03	Portuguese and Spanish	Pinto et al. [28]
		Parboiled	0.24/0.07	0.20/0.04		
		Wild	0.19/0.08	0.50/0.03		
		White	0.01/0.05	0.60/0.04		
PTWI [mg/week]	Hg	All rice products	0.0014	0.0014–0.0017	Chinese	Xu et al. [37]
THQ	As/Cd/Pb	Rinsed (white)	0.0230/0.0017/0.0005	0.0739–0.1855/ 0.0083–0.0207/ 0.0009–0.0024	Iranian	Shariatifar et al. [38]
		Traditionally cooked (white)		0.2883–0.6883/ 0.0116–0.0277/ 0.0014–0.0034		
THQ	As/Cd	Rice A (white) Rice B (brown) Rice C (white) Rice D (white)	0.0487/0.0017 (brown) 0.0230/0.0017 (white)	0.425/0.027 2.79/0.131 1.65/0.035 3.58/0.057	Thai	Kukusamude et al. [32]
HI	As, Cd, Pb	Rinsed (white)	0.0259	0.1053–0.2654	Iranian	Shariatifar et al. [38]
		Traditionally cooked (white)		0.3147–0.7522		

* As in total, EDI—estimated daily intake, EWI—estimated weekly intake, PTWI—provisional tolerable weekly intake, THQ—target hazard quotient, HI—hazard index.
